# A Low-Noise-Level Heart Sound System Based on Novel Thorax-Integration Head Design and Wavelet Denoising Algorithm

**DOI:** 10.3390/mi10120885

**Published:** 2019-12-17

**Authors:** Shuo Zhang, Ruiqing Zhang, Shijie Chang, Chengyu Liu, Xianzheng Sha

**Affiliations:** 1 School of Fundamental Sciences, China Medical University, Shenyang 110122, China; zs_techo@163.com (S.Z.);; 2 School of Instrument Science and Engineering, Southeast University, Nanjing 210096, China

**Keywords:** flexible electric film, wavelet denoising, digital stethoscope

## Abstract

Along with the great performance in diagnosing cardiovascular diseases, current stethoscopes perform unsatisfactorily in controlling undesired noise caused by the surrounding environment and detector operation. In this case, a low-noise-level heart sound system was designed to inhibit noise by a novel thorax-integration head with a flexible electric film. A hardware filter bank and wavelet-based algorithm were employed to enhance the recorded heart sounds from the system. In the experiments, we used the new system and the 3M™ Littmann® Model 3200 Electronic Stethoscope separately to record heart sounds in different noisy environments. The results illustrated that the average estimated noise ratio represented 21.26% and the lowest represented only 12.47% compared to the 3M stethoscope, demonstrating the better performance in denoising ability of this system than state-of-the-art equipment. Furthermore, based on the heart sounds recorded with this system, some diagnosis results were achieved from an expert and compared to echocardiography reports. The diagnoses were correct except for two uncertain items, which greatly confirmed the fact that this system could reserve complete pathological information in the end.

## 1. Introduction

Cardiovascular diseases (CVDs) are responsible for about 17.7 million deaths every year, representing 31% of global mortality [1]. Heart sounds are significant for screening toward CVDs, so research in this field such as the PhysioNet/Computing in Cardiology (CinC) Challenge 2016 [2] has received great attention recently. Combined with its noninvasive and cost-effective characteristics, the stethoscope is regarded as the standard equipment to detect heart sounds and has played an irreplaceable role in clinical practice [3]. 

The stethoscope was invented by René Laennec in France in 1816 [4], and the first article relevant to the stethoscope was published by Golding Bird [5]. Stethoscope technology has gradually matured with the developments over nearly 200 years [6,7,8,9]. However, undesired noise always interferes with doctors in getting accurate diagnoses. Meanwhile, plenty of attempts have been made to improve this. For example, Belloni et al. [10] applied multiple sensors for noise cancellation; different filters were used by several investigators to improve the signal-to-noise ratio. Jatupaiboon et al. [11] and Ghavami et al. [12] used adaptive noise reduction and adaptive line enhancement, whereas different filtering techniques using the least-mean squares, recursive least-squares algorithm, and linear prediction using autoregression were discussed by Gnitecki and Moussavi [13].

Although denoising research has made much progress, the noise cancelling of stethoscopes is still challenging as the problem of in-band noise comes from recorded heart sounds [14,15]. Besides, the noise-reducing methods mentioned above were mostly designed for ambient noise, and the inhibition of operating noise caused by the diaphragm’s relative motion with the thorax, clothes, or fingers [14] was unreasonably neglected. In our work, a low-noise-level heart sound system is designed. This system is able to eliminate both the ambient and operating noises and has proved to be more qualified compared to state-of-the-art digital stethoscopes.

The paper is organized as follows: the structure of the novel head and hardware design is provided in Section 2.1; algorithms for further denoising are provided in Section 2.2; the experiments are described in Section 3; results are presented in Section 4; discussions for results, limitations, and prospects are provided in Section 5; and the conclusions are drawn in Section 6.

## 2. Methods 

**Figure 1:**
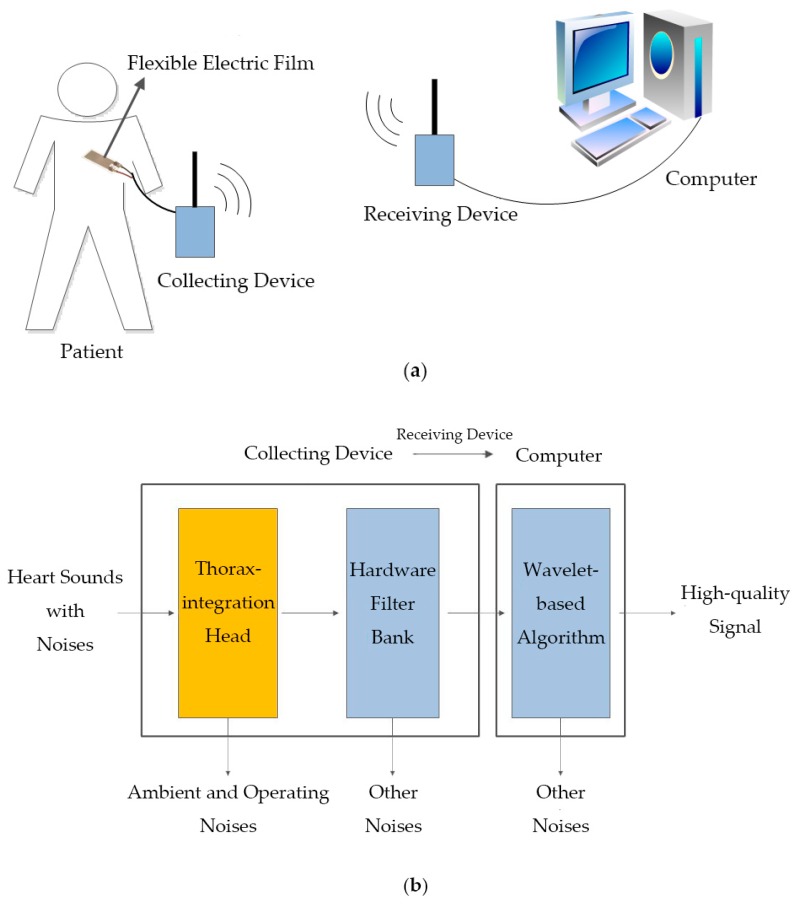
Architecture of the low-noise-level heart sound system: (**a**) operational schematic diagram; (**b**) denoising procedure diagram.

Shown in Figure 1a, the system was composed of two major parts: portable collecting and receiving devices and a wavelet-based algorithm. As for denoising characteristics (shown in Figure 1b), ambient and operating noises were first eliminated by a novel thorax-integration head, and some other noises were further suppressed by a hardware filter bank and wavelet-based algorithm.

### 2.1. Collecting and Receiving Devices

#### 2.1.1. Novel Thorax-Integration Head

**Figure 2:**
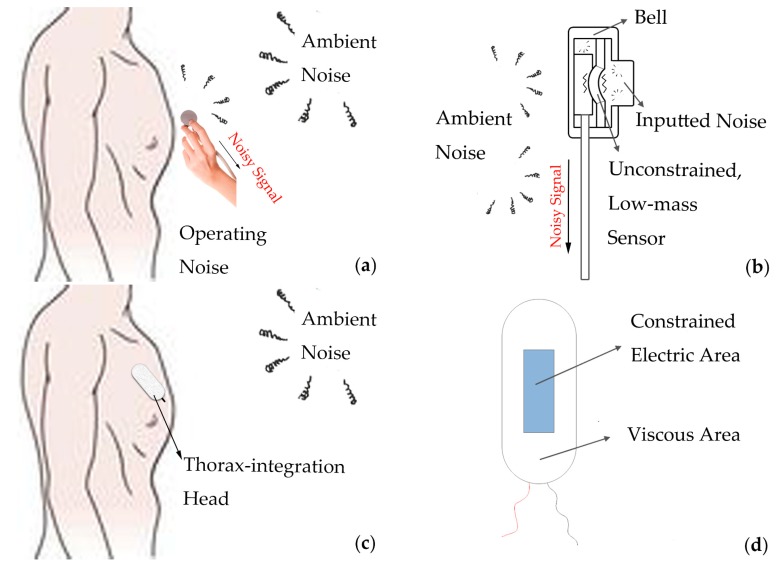
Diagram of traditional “Bell” head and novel thorax-integration head: (**a**) operation of “Bell” head; (**b**) structure of “Bell” head; (**c**) operation of thorax-integration head; (**d**) structure of thorax-integration head.

Firstly, we illustrate why heart sounds from traditional stethoscopes are noisy. On the one hand, due to its limited “Bell” structure, the stethoscope head has to be manually pressed on the thorax during detector operation, but this “unstable” contact probably results in operating noise caused by the diaphragm’s relevant motion with the thorax, clothes, or fingers (shown in Figure 2a). On the other hand, the low-mass sensor in the hollow-structure head is vibration-unconstrained, and the ambient noise with even a weak represented energy is always able to vibrate it to produce an undesired noisy signal (shown in Figure 2b).

Based on the above drawbacks, a novel head was designed (shown in Figure 2c–d). It was composed of two main parts: constrained transformation area converting the sounds into voltage signals and viscous area sticking the head to the thorax. Due to this thorax-integration structure, the vibration of the chest caused by heart sounds can be completely collected, but, remarkably, the energy of ambient noise was no longer enough to vibrate the sensor constrained by the thorax, which can inhibit the ambient noise. Moreover, this “stable” contact without hand-assistance avoided the operating noise to the largest extent during the measurement. Besides, the viscosity of the head was suitable to conveniently change the interested areas, and the viscous area could be completely replaced to meet different people’s demands.

**Figure 3:**
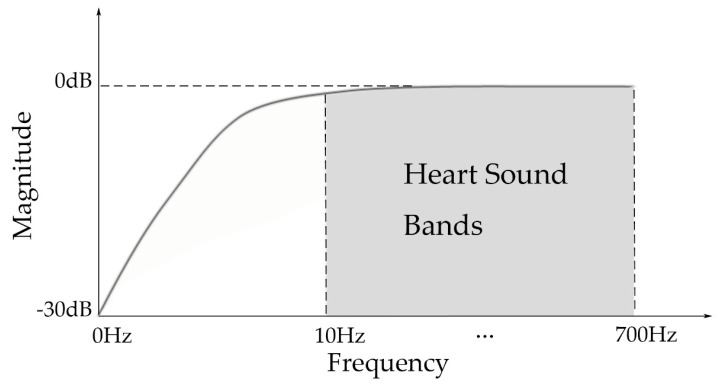
Frequency response of the electric area.

Complementally, the high-sensitivity transformation area was piezoelectric. According to its frequent responses shown in Figure 3, the curve of the used sensor was flat and stable in the heart sound band (10–700 Hz as usual), which revealed the low distortion of the collected signals. 

**Figure 4:**
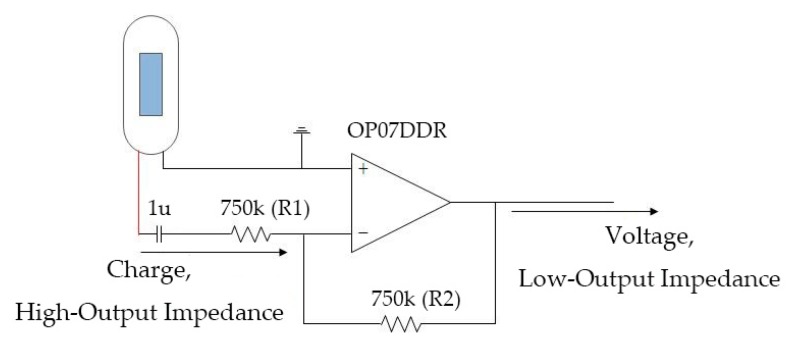
Schematic diagram of the charge amplifier.

However, the hardware design of the piezo-based device was more complicated. A charge amplifier was applied to transform the high-output impedance of the piezoelectric sensor. Specifically, it is necessary to adjust values of the resistances (R1 and R2) to change the input impedance and gain, and to obtain a voltage signal with a low-output impedance to process further (shown in Figure 4). 

#### 2.1.2. Hardware Filter Bank

**Figure 5:**
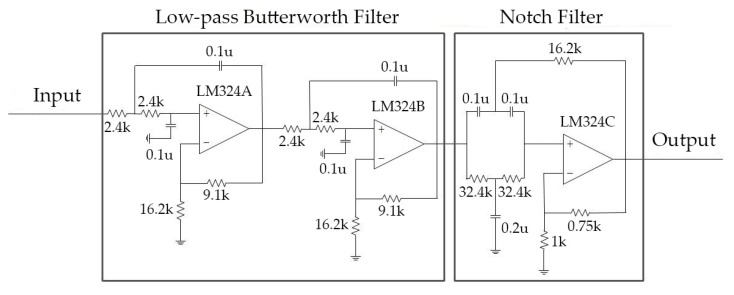
Circuit diagram of the hardware filter bank.

Further, a hardware filter bank (shown in Figure 5) was applied to remove other noise, which can be distinguished by frequency. Specifically, the signal was first input into a low-pass 4th order Butterworth filter to remove components out of the heart sound band. Then, a parallel-T RC notch filter was applied to inhibit the power-line frequency interferences.

#### 2.1.3. Other Hardware Design

**Figure 6:**
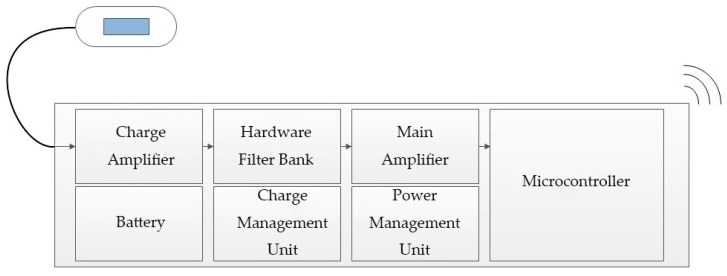
Schematic diagram of the collecting device.

Five other modules were present in the collecting device (shown in Figure 6): a main amplifier was applied to enlarge the filtered signal. A microcontroller called CC2530 was applied to convert the analog signals to digital signals and implement wireless transmission. Meanwhile, the collecting device was supplied by a chargeable battery of 5 V and converted to ±3.3 V through the power management unit. All key electronic components used in the collecting device are shown in Table 1.

As for the receiving device, CC2530 was also adopted as the core. The digital signal was received wirelessly and sent to the computer software from USART for further denoising. 

### 2.2. Wavelet-Based Denoising Algorithm

A wavelet-based algorithm was introduced to further inhibit noise. Wavelet transform (WT) is the most commonly used method for feature extraction, which can decompose heart sound signals using different mother functions such as ‘db10’ [16,17,18], ‘bior5.5’ [16,19,20], and ‘db5’ [19]. We briefly introduce several notations and definitions that will be used throughout this paper.

#### 2.2.1. Discrete Wavelet Transform

The wavelet transform is a mathematical analysis tool for signals. Its representation involves the decomposition of the signals in the mother wavelet functions given as [21]

(1) $$\psi_{a,b}\left(t\right)=\frac{1}{\sqrt{a}}\psi \left(\frac{t-b}{a}\right),for\;a,b\in R,$$

where a,b are the scale and position parameters, respectively. If the scales and positions are selected based on powers of two, so-called Dyadic scales and positions, then analysis becomes much more efficient and just as accurate. In this case, the wavelet function becomes [21]

(2) $$\psi_{m,n}\left(k\right)=2^{-\frac{m}{2}}\psi \left(2^{-m}k-n\right),for\;m,n\in Z.$$

In an orthonormal basis for $L^{2}\left(R\right)$, for a given function f(k), the inner product $\langle f,\psi_{m,n}\rangle$ then gives the discrete wavelet transform as [21,22]

(3) $$DWT\left(m,n\right)=\;\langle f,\psi_{m,n}\rangle \;=\;2^{-\frac{m}{2}}\sum_{k=-\infty }^{\infty }f\left(k\right)\psi^{*}\left(2^{-m}k-n\right).$$

#### 2.2.2. Hard Thresholding

Hard thresholding, based on establishing to zero the coefficients whose absolute values are less than the threshold, otherwise the coefficient value is not modified [23], is given as

(4) $$f_{H}=\left\{\begin{array}{c} C\left(n\right),for\left|C\left(n\right)\right|\geq T\\ 0,Otherwise \end{array}\right\},$$

where C(n) represents the coefficients and T the thresholding value [23].

#### 2.2.3. Soft Thresholding

Soft thresholding, based on establishing to zero the coefficients whose absolute values are less than the threshold, otherwise the coefficient value is equal to the value of a sign function that multiplies the subtraction value between the coefficient and threshold T [23], is given as

(5) $$f_{S}=\left\{\begin{array}{c} sgn\left(C\left(n\right)\right)\left(C\left(n\right)-T\right),for\left|C\left(n\right)\right|\geq T\\ 0,Otherwise \end{array}\right\}.$$

The sign function returns 1, 0, or −1 if the coefficient is greater than zero, equal to 0, or less than zero, respectively [23].

#### 2.2.4. Minimax Thresholding Techniques

Thresholding selection rules consist of mathematical calculations that can provide a representative noise threshold. The minimax selection rule uses a fixed threshold to produce a minimax performance for the root-mean-square error against an ideal procedure [24], given as

(6) $$th_{j}=\left\{\begin{array}{c} \sigma \left(0.3936+0.10829\;\log_{2}N\right),N>32\\ 0,N\leq 32 \end{array}\right\},\;$$

where $\sigma =\mathrm{median}\left(\frac{\omega }{0.6745}\right)$, ω is regarded as the wavelet coefficient vector at the unit scale, and N is the length of the signal vector [24].

#### 2.2.5. Denoising Procedure

**Figure 7:**
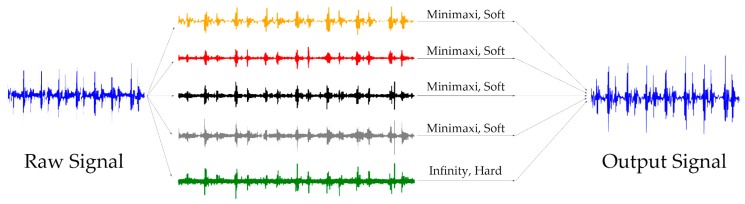
Procedure of wavelet-based denoising algorithm.

As it is shown in Figure 7, the raw signal was first decomposed with level 4 using the ’db10’ mother wavelet. The components of 5 frequency bands of 0–62.5, 62.5–125, 125–250, 250–500, and 500–1000 Hz were obtained. Further, as the last component was over the frequency band of heart sounds, the hard thresholding method with an infinite threshold was selected to eliminate this part. Then, the soft thresholding method with the minimax threshold was selected to denoise other components. Finally, we reconstructed the processed coefficient matrixes and obtained the output signal.

## 3. Experiment

### 3.1. Denoising Experiment

**Figure 8:**
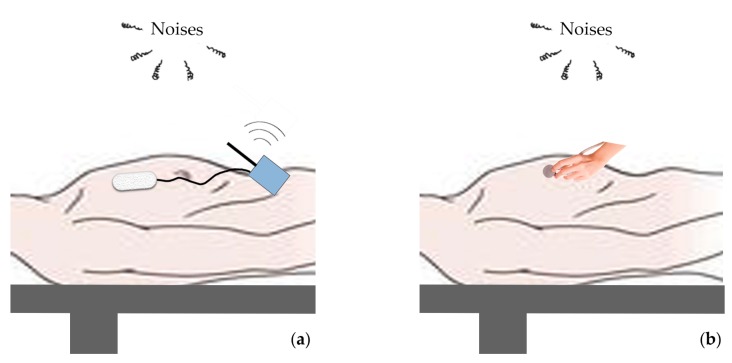
Schematic diagram of experiment’s operation: (**a**) operation of proposed system; (**b**) operation of 3M™ Littmann® Model 3200 Electronic Stethoscope.

We used the new system and the 3M™ Littmann® Model 3200 Electronic Stethoscope separately to record heart sounds in several noisy environments to test its performances in denoising. Specifically, a volunteer was arranged to lie down and some noises containing knocking, cheering, typing, clapping, and striking were made to interfere with the collection of heart sounds. Then, two equipment above were applied to record 15 s signals of heart sounds (shown in Figure 8). It was shown that the relative distance between the noisy source, equipment, and the energy of noises remained unchanged during the measurement.

Next, we further illustrate their performances with the normalized standard deviation (STD) criteria [25]. Specifically, each signal was first normalized to avoid the influence of different magnifications of the equipment, and was then averagely segmented to five frames. In the following steps, the fast Fourier transform (FFT) was applied in each frame. The procedure of calculating is given as

(7) $$\bar{x_{t}}\left(l\right)=\frac{1}{K}\overset{K}{\underset{k=1}{\sum }}{\left|X\left(k,l\right)\right|}^{2},$$

(8) $$v_{t}\left(l\right)=\sqrt{\left[\frac{1}{K}\overset{K}{\underset{k=1}{\sum }}{\left({\left|X\left(k,l\right)\right|}^{2}-\bar{x_{t}}\left(l\right)\right)}^{2}\right]},$$

(9) $${{\hat{\sigma }}_{t}}^{2}=\frac{1}{L}\overset{L}{\underset{l=1}{\sum }}v_{t}\left(l\right),$$

where X(k,l) represents the coefficient vector of the signal, k (k=1, 2,…, 30,000) represents the frequency bin index, l (l=1, 2,…, 5) represents the frame index, $\bar{x_{t}}\left(l\right)$ represents the average noise power spectrum in the frequency bin, and ${{\hat{\sigma }}_{t}}^{2}$ represents the assumed estimate of noise power [25]. Then, the estimate noise ratio (*ENR*) defined for comparing their characteristics in inhibiting noise is given as

(10) $$ENR=\frac{{\hat{\sigma }}^{2}{}_{Proposed\;System}}{{\hat{\sigma }}^{2}{}_{3M}}\times 100\%,$$

where ${\hat{\sigma }}^{2}{}_{Proposed\;System}$ represents the assumed estimate of noise power in the recording from the proposed system, and ${\hat{\sigma }}^{2}{}_{3M}$ represents that from the 3M stethoscope.

### 3.2. Reserve Pathological Information Experiment

We implemented another test to illustrate whether this system could record complete pathological information. Specifically, an expert was invited to diagnose 10 recordings collected with the system. These recordings were composed of four abnormal audios representing rheumatic heart disease, the bicuspid aortic valve, after aortic valve replacement surgery, and aortic regurgitation, and 6 normal audios. Then, the final diagnosis results were compared to echocardiography reports that were regarded as the “golden standard” in the structural abnormality of the heart.

## 4. Results

### 4.1. The Established Prototype

**Figure 9:**
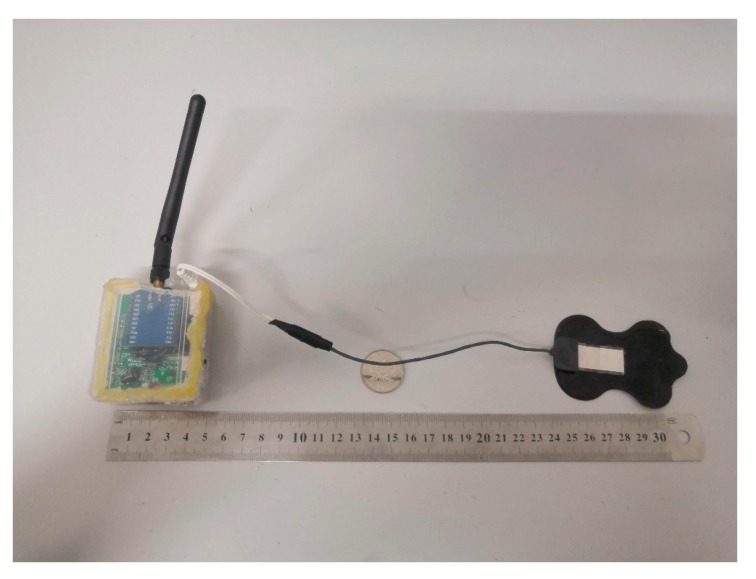
Image of established prototype.

The established prototype is shown in Figure 9 and the main technical parameters are shown in Table 2. Specifically, the prototype was tiny but effective. The sample rate of 2 kHz ensured the high fidelity of signals. Long battery life and short charging time further promoted its feasibility.

**Figure 10:**
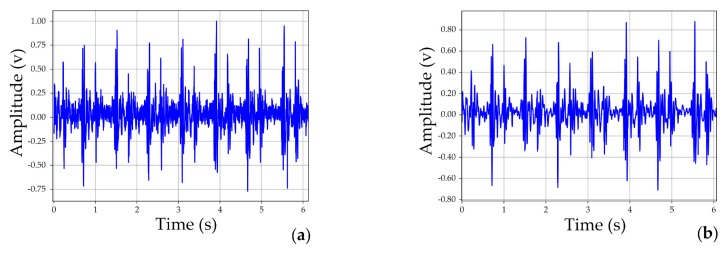
Collected signals using the low-noise-level heart sound system: (**a**) raw signal; (**b**) output.

As for the quality of signals, it is evident that the fundamental components: S1, systole, S2, diastole, were completely recorded in the raw signal (shown in Figure 10a), but some noise was inevitably observed. However, the output (shown in Figure 10b) represented a much purer signal.

### 4.2. Results of Experiments

#### 4.2.1. Results of Denoising Experiment

**Figure 11:**
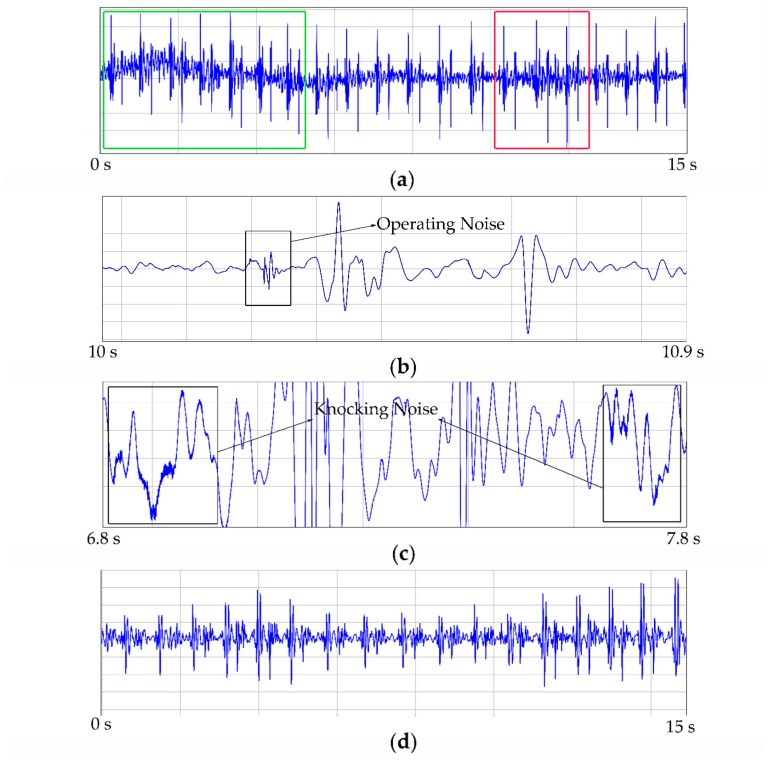
Recordings in denoising experiments: (**a**) recording from 3M™ Littmann® Model 3200 Electronic Stethoscope in knocking environment; (**b**) operating noise in (**a**); (**c**) ambient noise in (**a**); (**d**) recording from low-noise-level heart sound system in knocking environment.

First, signals collected in the knocking environment were abstracted as an example to specifically analyze its components. Shown in Figure 11, the recording from the 3M stethoscope (shown in Figure 11a) represented an apparent baseline shift in the green frame. The operating noise might have been heard in the whole audio, especially strong in the red frame. This recording was extended for further analysis. We noticed that the operating noise (shown in Figure 11b) was similar to those of S1 and S2, causing an obstacle to the filter. Moreover, knocking noises also always appeared in the background (shown in Figure 11c) and it seems that a high-frequency wave was attached to the envelope of heart sounds. However, the recording from the proposed system (shown in Figure 11d) was much purer and more stable. Analogously, the recordings from the system also had similar characteristics to other kinds of noise tests (cheering, typing et al.).

Furthermore, calculated by Equations (7)–(10) introduced in Section 3.2, estimate noise ratios in all noisy environments were lower than 100%, demonstrating that the recordings from the proposed system were indeed purer. The lowest appeared in the typing test, only representing 12.47%, while the highest appeared in the knocking, representing 59.05%. Moreover, the mean represented 21.26% (shown in Table 3). It is evident that the system had a better capability in inhibiting noise compared to the state-of-the-art stethoscope.

#### 4.2.2. Results of Reserve Pathological Information Experiment

**Figure 12:**
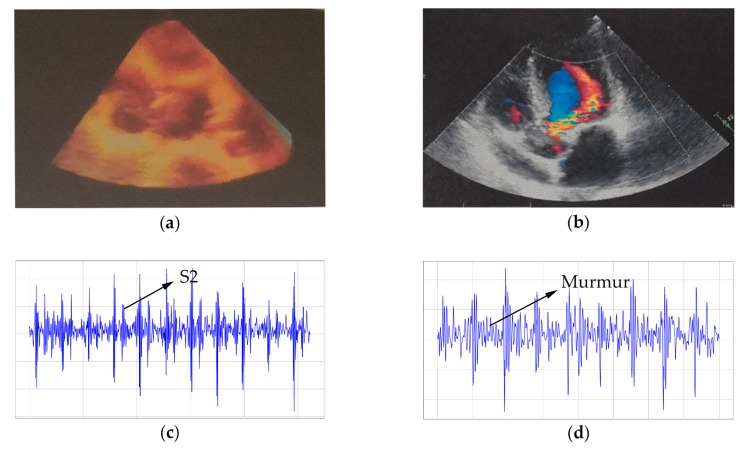
Normal/abnormal recordings: (**a**) and (**c**) represent the normal recording separately by echocardiography and the proposed system; (**b**) and (**d**) represent the abnormal recording separately by echocardiography and the proposed system.

As far as pathological information, fundamental heart sounds (such as S1 and S2) were obvious in the normal recording shown in Figure 12c, while S2 replaced by murmurs was in the abnormal recording shown in Figure 11d. Their corresponding echocardiography maps are shown in Figure 12a,b. Furthermore, except for two uncertain items, the rest of the diagnoses were correct in the expert’s report.

## 5. Discussion

In this study, a heart sound system was designed to solve the problem of noisy interference while detecting heart sounds. This system adopted a novel viscous electric film head to achieve thorax-integration measurement in order to validly suppress most of ambient noise and operating noise. Moreover, the introduced hardware filter bank and denoising algorithm also achieved an excellent performance in obtaining a purer signal afterward. 

### 5.1. The Excellent Capability of Thorax-Integration Head in Denoising

As the results have shown, the 3M stethoscope has not performed satisfactorily in denoising even with advanced methods. Usually, a microphone or piezoelectric sensor is applied in the traditional “Bell” head to collect a signal. For detecting the weak sounds coming from tiny lesions in the heart, the sensor is preferred to be minuscule and unconstrained, along with undesired noise being inevitably collected. Innovatively, the newly designed thorax-integration head can not only detect heart sounds through stably attaching to the chest but also through being constrained by the chest, which can perfectly solve the contradiction. The results also illustrate that this system has a better performance in denoising than the 3M stethoscope. Hence, the microphone is unsuitable to be a sensor in the new system due to its hollow structure.

### 5.2. Noise Components in Raw Signal

As said above, some other noises were still so apparent in the raw signal. With the consideration of the use of a notch filter in hardware processing, it may be mainly caused by analog-to-digital conversion (ADC). Specifically, ADCs mainly include two kinds of noise: quantization noise caused by the limited width of the digital signal and intrinsic noise (such as comparator noises) depending on the characteristic of the element [26]. On the one hand, the adopted ADC employing a 12-bit sensitivity may not enough to produce a high-frequency smooth digital signal, and some reparable methods such as signal-digital-averaging were absent in the proposed system. On the other hand, considering a balance of other modules in CC2530, its intrinsic noise should always be larger than that of the specific ADC chip. Besides, we expected a large transmitting power to satisfy a long-distance transmission. However, due to the imperfect design in electromagnetic shielding, the signal is easily interfered by the large transmitting power in the main amplifying procedure before digitization. The introduced denoising algorithm is essential to solve this problem. Specifically, this noise was stochastic and obeyed a normal distribution. Thresholding techniques can provide a suitable value to suppress most noise components. Due to this, the system could produce a high-quality signal while achieving a long-distance transmission of 70.4 m, nearly seven times that of the state-of-the-art stethoscope.

### 5.3. Complete Pathological Information in Its Recordings

It is true that some medical equipment must be applied to record pathological information to assist doctors in diagnosing, which is maybe more important than its denoising characteristic. As it is shown in the above results, an obvious murmur appeared in the abnormal recording (shown in Figure 12d), and the diagnosis of the expert was almost correct on the collected dataset, which demonstrates that the new system could completely reserve the pathological information in its recordings. Honestly, one "normal" report from the expert is different from the “golden standard” of echocardiography. However, the expert firmly believed that a premature beat appeared inside. Indeed, echocardiography only reflects the structural abnormality, which further produces most abnormal heart sounds, but some abnormal rhythms like premature beats could not be written in its report. We strongly believe that with the help of other experts, this recording is an exact premature beat with its further fidelity characteristic. As for the two uncertain recordings, the expert said that the energy of the heart sounds was too weak to obtain a precise diagnosis. It was mainly caused by the incorrect detector location. Specifically, all recordings were collected by our researchers who lacked physiological knowledge, and the collector of uncertain recordings revealed that their detector locations were over the sternum rather than the intercostal space, which led to the weak energy, so that obtained an uncertain diagnosis.

### 5.4. Limitations and Prospects

Limitations should be considered: first, although recordings can be broadcast through the computer, the lack of earphones in the collecting device causes users to not be able to directly hear patients’ heart sounds, which limits the convenience of the system. Secondly, the poor electromagnetic shielding characteristic causes the collecting device to perform badly in resisting the electromagnetic interference. Thirdly, the signal is too weak when the head is attached to sternums. We plan to further perfect it in future work.

Besides, the automated analysis in heart sounds is significant in the clinical research, so the 2016 CinC Challenge [2] has focused on this field and encouraged the development of algorithms for accurate classification [27,28,29]. In the future, some artificial intelligence technologies will also be applied in the system to automatically analyze big heart sound data and provide auxiliary diagnosis functions for clinicians. In addition, a more effective chip will be employed in the collecting device to act as the hardware implementation of the introduced wavelet-based method.

## 6. Conclusions

In this study, a new heart sound system with a novel thorax-integration head and wavelet-based algorithm was introduced to solve problems of noise interference during the heart sound detection procedures. The experimental results have illustrated that the system is able to record complete pathological information with assistance of an expert who has achieved mostly correct diagnoses in the collected dataset. Moreover, it obtained purer signals with only an average of 21.26% noise level compared to the 3M stethoscope. In conclusion, the new system has demonstrated its better performance and accuracy compared to state-of-the-art digital stethoscopes.

## Figures and Tables

**Table 1:** Key electronic components used in collecting device.

Item	Manufacturer	Chip
Preamplifier	Texas Instrument	OP07DDR
Filter bank	Texas Instrument	LM324DR
Main amplifier	Texas Instrument	OP07DDR
Charge management unit	H&Msemi	HM4061
Power management unit	Suosemi Techology, Maxim Intergrated	SX1308, MAX660
Microcontroller	Texas Instrument	CC2530

**Table 2:** Main technical parameters of the collecting prototype.

Size (mm)	Weight (g)	Sampling Rate (Hz)	Battery Life (h)	Charging Time (h)
65 × 50 × 25	73.4	2000	18–20	3–4

**Table 3:** Estimated noise ratio in different noise tests.

Noises	Knocking	Cheering	Typing	Clapping	Striking	Mean
*ENR*	59.05%	17.43%	12.47%	41.94%	17.44%	21.26%
